# Effects of Elevated CO_2_ on Levels of Primary Metabolites and Transcripts of Genes Encoding Respiratory Enzymes and Their Diurnal Patterns in *Arabidopsis thaliana*: Possible Relationships with Respiratory Rates

**DOI:** 10.1093/pcp/pct185

**Published:** 2014-01-18

**Authors:** Chihiro K. Watanabe, Shigeru Sato, Shuichi Yanagisawa, Yukifumi Uesono, Ichiro Terashima, Ko Noguchi

**Affiliations:** ^1^Department of Biological Sciences, Graduate School of Science, The University of Tokyo, 7-3-1 Hongo, Bunkyo-ku, Tokyo, 113-0033 Japan; ^2^Laboratory of Proteome Research, Proteome Research Center, National Institute of Biomedical Innovation, 7-6-8 Saito-Asagi, Ibaraki, Osaka, 567-0085 Japan; ^3^Biotechnology Research Center, The University of Tokyo, 1-1-1 Yayoi, Bunkyo-ku, Tokyo, 113-8657 Japan

**Keywords:** A*rabidopsis thaliana*, Elevated CO_2_, Primary metabolites, Respiration, Transcription

## Abstract

Elevated CO_2_ affects plant growth and photosynthesis, which results in changes in plant respiration. However, the mechanisms underlying the responses of plant respiration to elevated CO_2_ are poorly understood. In this study, we measured diurnal changes in the transcript levels of genes encoding respiratory enzymes, the maximal activities of the enzymes and primary metabolite levels in shoots of *Arabidopsis thaliana* grown under moderate or elevated CO_2_ conditions (390 or 780 parts per million by volume CO_2_, respectively). We examined the relationships between these changes and respiratory rates. Under elevated CO_2_, the transcript levels of several genes encoding respiratory enzymes increased at the end of the light period, but these increases did not result in changes in the maximal activities of the corresponding enzymes. The levels of some primary metabolites such as starch and sugar phosphates increased under elevated CO_2_, particularly at the end of the light period. The O_2_ uptake rate at the end of the dark period was higher under elevated CO_2_ than under moderate CO_2_, but higher under moderate CO_2_ than under elevated CO_2_ at the end of the light period. These results indicate that the changes in O_2_ uptake rates are not directly related to changes in maximal enzyme activities and primary metabolite levels. Instead, elevated CO_2_ may affect anabolic processes that consume respiratory ATP, thereby affecting O_2_ uptake rates.

## Introduction

The atmospheric CO_2_ concentration has drastically increased in recent decades, and it is expected to increase to double the level that it was before the industrial revolution during this century. Plants fix approximately 150–175 Pg carbon (C) per year via photosynthesis (DeLucia et al. 2007, Welp et al. 2011), and release about half of the fixed C via respiration (Lambers et al. 2005). The amount of C released by terrestrial plant respiration was estimated to be approximately 6-fold that released by anthropogenic activities (Amthor 1995, Canadell et al. 2007, Houghton 2007). Photosynthetic responses to elevated CO_2_ have been well studied. In most of the C_3_ species examined so far, the photosynthetic rate on a leaf area basis increases under elevated CO_2_, although the extent of the increase varies among species and among different experimental conditions (Long et al. 2004, Leakey et al. 2009a). Meta-analyses have shown that respiratory rates on a leaf area basis generally increase under elevated CO_2_ (Wang and Curtis 2002, Leakey et al. 2009a); this is partly attributed to a greater leaf mass per area (LMA) in plants grown under elevated CO_2_. The respiratory rates on a mass basis are often lower in plants grown under elevated CO_2_ than in those grown under ambient CO_2_ (Wang and Curtis 2002, Gonzalez-Meler et al. 2004). However, the mechanisms underlying changes in the respiratory rate under elevated CO_2_ remain unclear.

The respiratory rate in plants is generally limited by substrate availability, enzyme capacity or the ATP consumption rate (Lambers et al. 2005). Under elevated CO_2_, growth and carbohydrate accumulation are enhanced in most plants (Long et al. 2004, Leakey et al. 2009b). These changes would alter the availability of respiratory substrates and/or the ATP consumption rate, leading to changes in the respiratory rate. Carbohydrate accumulation would also affect the transcript levels of genes encoding respiratory enzymes, which would affect the amounts of their encoded products. The levels of carbohydrates, primary metabolites and gene transcripts show substantial diurnal changes (Blasing et al. 2005, Gibon et al. 2006), which may be responsible for diurnal changes in the respiratory rate (Noguchi 2005). Therefore, the effects of elevated CO_2_ on diurnal changes in the levels of primary metabolites, gene transcripts and the capacity of enzymes in the respiratory system should be examined to identify which factors limit the respiratory rate under elevated CO_2_.

Previous studies showed that the transcript levels of many genes encoding respiratory enzymes are up-regulated in plants grown under elevated CO_2_, compared with those in plants under ambient CO_2_ (Li et al. 2008, Leakey et al. 2009b). The number of mitochondria also increases under elevated CO_2_ (Griffin et al. 2001, Wang et al. 2004). Tissue et al. (2002) reported that the number of mitochondria per cell area was significantly greater, but the activity of the cytochrome *c* oxidase (COX) was not affected in leaves of sweetgum (*Liquidambar styraciflua*) grown under elevated CO_2_. In contrast, the activities of succinate dehydrogenase and COX in cotyledons of soybean were lower in plants grown under elevated CO_2_ than in those grown under ambient CO_2_ (Gonzalez-Meler et al. 1996). Changes in transcript levels do not always correlate with changes in enzyme capacities; therefore, it is important to investigate both transcript levels and the activities of respiratory enzymes. To date, however, such studies have not been reported.

In a previous study, plants grown under elevated CO_2_ showed increased levels of not only carbohydrates, but also various intermediates of the respiratory system such as sugars and organic acids (Li et al. 2008). Since these increases reflect changes in the availability of respiratory substrates, they should affect the respiratory rate. However, it is unclear whether changes in the levels of primary metabolites directly affect the respiratory rate under elevated CO_2_. Also, the changes in organic acid levels may result in different O_2_ uptake and CO_2_ efflux rates, because accumulation and consumption of organic acids affect the respiratory quotient (RQ) (Lambers et al. 2005).

Plant growth is often enhanced under elevated CO_2_, and the amount of carbohydrates exported from leaves increases (Long et al. 2004). These changes would enhance the consumption rate of respiratory ATP, leading to changes in the respiratory rate. However, it is still unknown whether changes in the consumption rate of respiratory ATP result in changes in the respiratory rate in response to elevated CO_2_.

The aim of this study was to examine the responses of plant respiration to elevated CO_2_. Our objectives were as follows: (i) to compare changes in the transcript levels of genes encoding respiratory enzymes in plants grown under elevated CO_2_ with those in plants grown under moderate CO_2_, and determine whether changes in gene transcript levels correspond to changes in activities of the enzymes they encode; (ii) to determine which primary metabolites accumulate under elevated CO_2_; and (iii) to evaluate whether the limiting factor for respiratory rates changes under elevated CO_2_. We used *Arabidopsis thaliana* because of its rapid growth rate, its strong growth response to elevated CO_2_ and because there is a vast array of -omics data available for this species (Li et al. 2006, Li et al. 2008). We grew *A. thaliana* under moderate or elevated CO_2_ conditions [390 or 780 parts per million by volume (ppmv) CO_2_, respectively] and analyzed whole shoots collected on day 20 after germination. At this time, most of the leaves on the shoot were expanding (Supplementary Fig. S1). Since growth rates are generally well correlated with respiratory rates (Reich et al. 1998), this material was suitable for investigating the responses of respiration to elevated CO_2_.

We measured the transcript levels of genes encoding enzymes involved in glycolysis, the tricarboxylic acid (TCA) cycle and the respiratory chain using real-time PCR, and measured the activities of several respiratory enzymes by spectrophotometric methods. We quantified some carbohydrates, amino acids and primary metabolites using spectrophotometry or capillary electrophoresis–mass spectrometry (CE-MS). The experimental materials were whole shoots sampled at three different times of the day. We measured the rates of CO_2_ efflux and O_2_ uptake to determine the respiratory rates in the shoots at the ends of both the light and dark periods. We also determined the effects of adding sucrose or an uncoupler on O_2_ uptake to determine whether the O_2_ uptake rate was limited by respiratory substrate availability or ATP consumption rates in the shoots. We analyzed these diurnal changes and discussed how elevated CO_2_ affects respiratory rates.

## Results

### Response of shoot growth to elevated CO_2_

The shoot DW of plants was significantly higher under elevated CO_2_ than under moderate CO_2_ (Supplementary Table S1). The relative growth rate (RGR) of shoots grown under elevated CO_2_ was higher than that of shoots grown under moderate CO_2_, except on day 28. The largest difference in RGR between the two CO_2_ treatments was on day 20, during the vegetative growth stage (Supplementary Table S2). Based on the change in leaf size between day 16 and day 24, most true leaves were expanding on day 20 (Supplementary Fig. S1). Therefore, the shoot on day 20 was considered as suitable experimental material to study the growing plant body, although the cotyledons had stopped expanding at this stage (Supplementary Fig. S1). In this study, the data are expressed on a FW basis except for transcript level data; the transcript levels of genes encoding respiratory enzymes are based on total RNA. This is because the efficiency of RNA extraction changed depending on starch accumulation in the leaves. We cultivated *A. thaliana* plants on plates (Supplementary Fig. S1) because plant growth was uniform and it was easy to harvest shoots for sampling.

### Responses of transcript levels and maximal activities of respiratory enzymes to elevated CO_2_

**Fig. 1:**
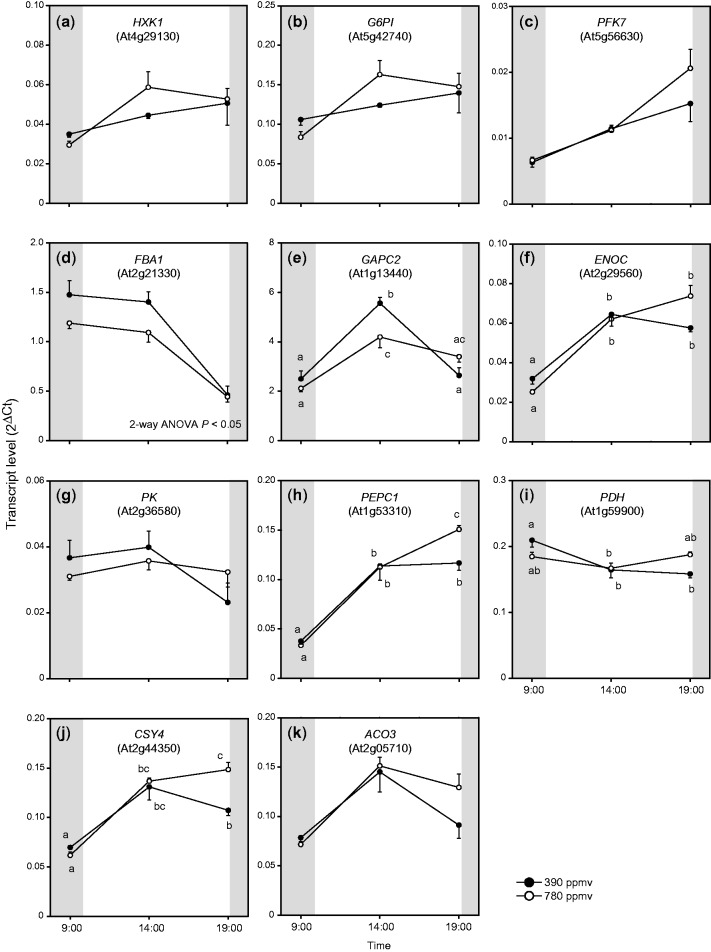
Diurnal changes in transcript levels of nuclear-encoded genes related to glycolysis and the TCA cycle in *A. thaliana* shoots. Transcript levels were calculated from the difference in threshold cycle (*Ct*) between *Rps15aA* and each gene. Mean values ± SEM are shown (*n* = 4). Three time points on the *x*-axis denote sampling times: end of the night period (09:00–10:00 h), middle of the day (14:00–15:00 h) and end of the light period (from 19:00 to 20:00 h). Two-way ANOVA was performed using CO_2_ treatment and sampling time as factors. *P*-value indicates significance of differences in transcript levels between elevated and moderate CO_2_. Other results of two-way ANOVA are shown in Supplementary Table S4. If two-way ANOVA interaction (*F*_time_ × _CO2_) was significant (*P* < 0.05), results of Tukey–Kramer’s multiple comparison tests are shown as different letters.

**Fig. 2:**
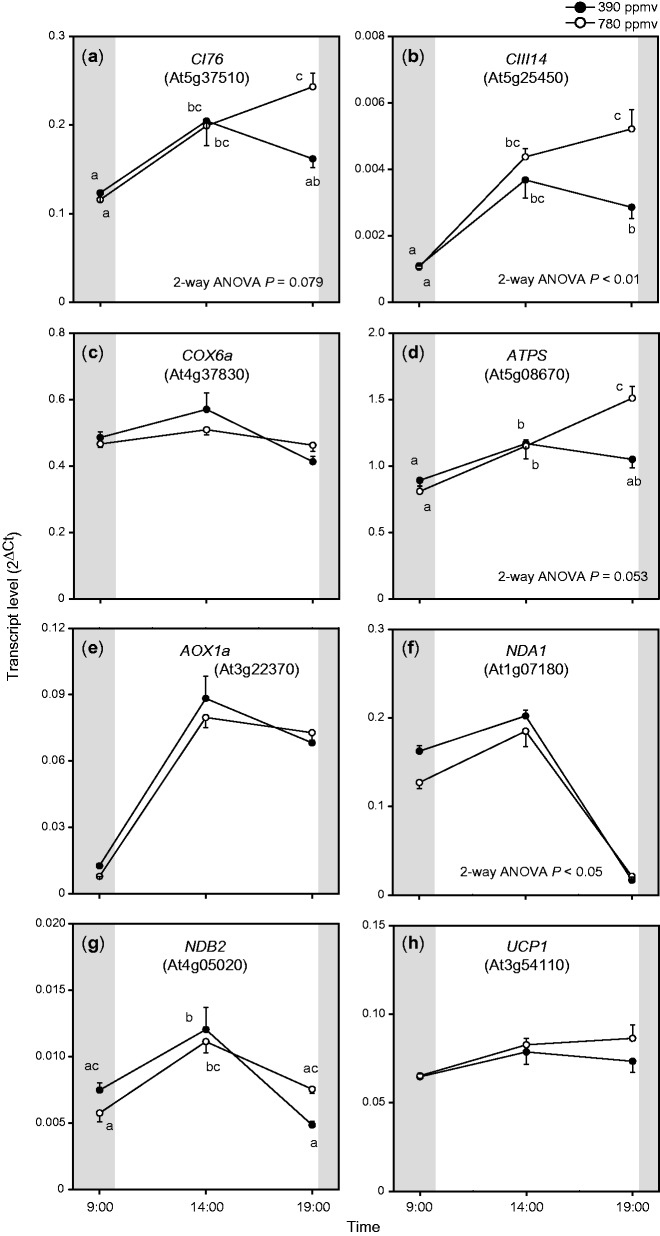
Diurnal changes in transcript levels of nuclear-encoded genes related to the respiratory chain in *A. thaliana* shoots. For other details, see the legend of Fig. 1.

We evaluated whether the transcript levels of genes encoding respiratory enzymes were increased in shoots of *A. thaliana* grown under elevated CO_2_ (Figs. 1, 2). We analyzed the transcript levels of nuclear-encoded genes related to glycolysis, the TCA cycle and the respiratory chain by real-time PCR. For each gene, the transcript level was calculated from the difference in the threshold cycle (*Ct*) between the gene and *Rps15aA*, the internal standard. We evaluated the transcript levels of eight genes related to glycolysis; *HXK1* (encoding hexokinase 1), *G6PI* (encoding glucose-6-phosphate isomerase), *PFK7* (encoding phosphofructokinase 7), *FBA1* (encoding fructose-1,6-bisphosphate aldolase 1), *GAPC2* (encoding glyceraldehyde-3-phosphate dehydrogenase C2), *ENOC* (encoding cytosolic enolase), *PK* (encoding pyruvate kinase) and *PEPC1* (encoding phospho*enol*pyruvate carboxylase 1). The transcript levels of some genes showed similar patterns. The transcript levels of *HXK1* and *G6PI* gradually increased during the day (Fig. 1a, b). The changes in transcript levels of *ENOC* and *PEPC1* were also similar, and their transcript levels at the end of the light period were higher under elevated CO_2_ than under moderate CO_2_ (Fig. 1f, h). In contrast, the transcript levels of *FBA1* and *GAPC2* were lower under elevated CO_2_ than under moderate CO_2_ (Fig. 1d, e).

We analyzed the transcript levels of genes encoding pyruvate dehydrogenase E1 alpha subunit (*PDH*), and citrate synthase 4 (*CSY4*) and aconitase 3 (*ACO3*) in the TCA cycle. *CSY4* and *ACO3* showed patterns similar to those of *ENOC* and *PEPC* (Fig. 1j, k); their transcript levels at the end of the light period were higher under elevated CO_2_ than under moderate CO_2_. The transcript level of *PDH* was slightly higher under moderate CO_2_ than under elevated CO_2_ at the end of the dark period, and slightly lower under moderate CO_2_ than under elevated CO_2_ at the end of the light period (Fig. 1i).

We analyzed the transcript levels of genes involved in the respiratory chain (Fig. 2). Three genes encoded components of phosphorylating pathways; the 76 kDa subunit of complex I (*CI76*), the 14 kDa subunit of complex III (*CIII14*) and the 6a subunit of cytochrome *c* oxidase (*COX6a*), and three genes encoded components of non-phosphorylating pathways; the alternative oxidase 1a (*AOX1a*), the type II NAD(P)H dehydrogenase A1 (*NDA1*) and B2 (*NDB2*). We also evaluated the transcript levels of genes encoding H^+^-ATPase (*ATPS*) and the uncoupling protein 1 (*UCP1*). At the end of the light period, the transcript levels of *CI76*, *CIII14* and *ATPS* were significantly higher under elevated CO_2_ than under moderate CO_2_ (Fig.2a, b, d). These diurnal patterns were similar to those of *ENOC*, *PEPC1*, *CSY4* and *ACO3*. The transcript levels of *AOX1a*, *NDA1* and *NDB2* were highest in the middle of the day. The expression of *NDA1* differed between the elevated and moderate CO_2_ treatments. There were lower transcript levels of *NDA1* under elevated CO_2_ than under moderate CO_2_. The transcript levels of *COX6a* and *UCP1* showed only small diurnal changes and were not significantly different between the two CO_2_ conditions. As summarized in these results, the transcript levels of some respiratory enzymes were higher under elevated CO_2_ than under moderate CO_2_, particularly at the end of the light period.

**Fig. 3:**
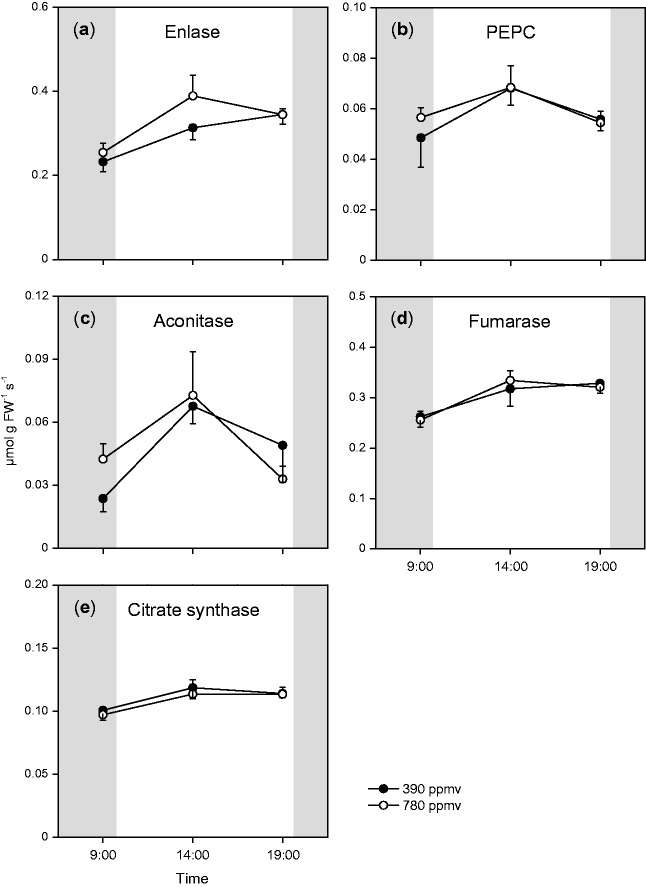
Diurnal changes in maximal activities of respiratory enzymes in *A. thaliana* shoots. Mean values ± SEM are shown (*n* = 5). For other details, see the legend of Fig. 1.

Next, we analyzed whether the increased transcript levels of respiratory enzymes affected the maximal activities of their corresponding respiratory enzymes under elevated CO_2_. We measured the activities of enolase and PEPC, which are involved in glycolysis, and citrate synthase, aconitase and fumarase, which function in the TCA cycle. The maximal activities of enolase, aconitase and fumarase showed some diurnal changes (Fig. 3), but there was no significant difference in the maximal activity of each enzyme between the two CO_2_ conditions during the day. Therefore, the increased transcript levels of genes encoding respiratory enzymes under elevated CO_2_ did not result in increased maximal activities of their corresponding enzymes in *A. thaliana* shoots.

### Changes in levels of primary metabolites under elevated CO_2_

**Fig. 4:**
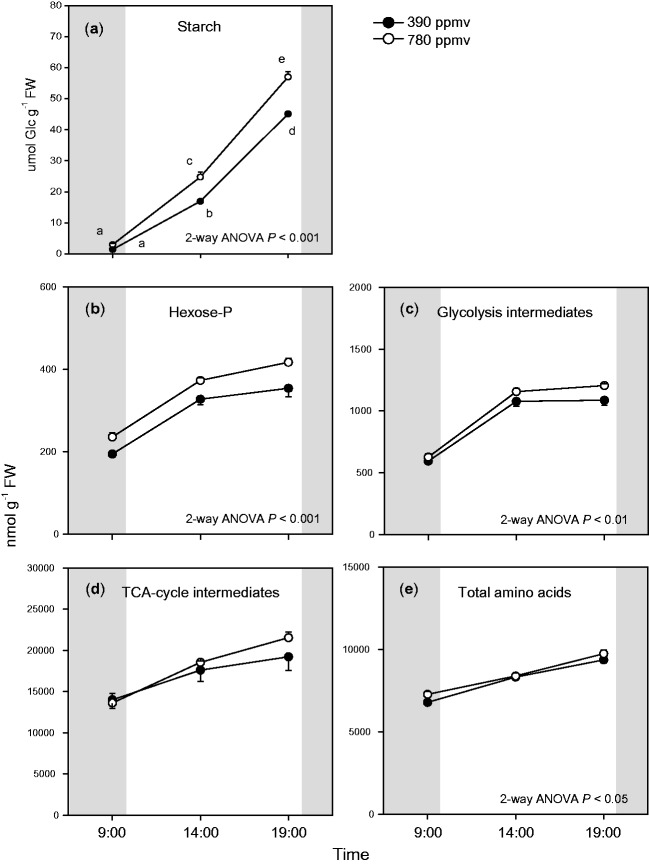
Diurnal changes in starch levels (a), total hexose phosphates (hexose-P) levels (b), total glycolysis intermediates levels (c), total TCA cycle intermediates levels (d) and total amino acid levels (e) in *A. thaliana* shoots. Hexose-P is the sum of levels of G1P, G6P, F6P and M6P. Glycolysis intermediates is the sum of levels of G6P, F6P, GAP, PGA, PEP and pyruvate. TCA cycle intermediates is the sum of levels of citrate, *cis*-aconitate, succinate, fumarate and malate. Mean values ± SEM are shown (*n* = 3–5). For other details, see the legend of Fig. 1.

The levels of carbohydrates and organic acids have been reported to increase in plants grown under elevated CO_2_ (Li et al. 2008, Leakey et al. 2009b). Therefore, we investigated which primary metabolites showed increased levels in *A. thaliana* shoots under elevated CO_2_. Since there were no differences in water contents [(FW – DW)/FW] of plants between the two CO_2_ conditions and among sampling times (data not shown), the changes in the levels of primary metabolites did not reflect changes in shoot water contents. We measured the levels of three non-structural carbohydrates: starch, glucose and sucrose. Starch accumulated during the day and the starch level was higher at under elevated CO_2_ than under moderate CO_2_ (Fig. 4a). The glucose and sucrose levels were lower than that of starch, with only small differences in glucose and sucrose levels between the two CO_2_ conditions (Supplementary Fig. S2).

**Fig. 5:**
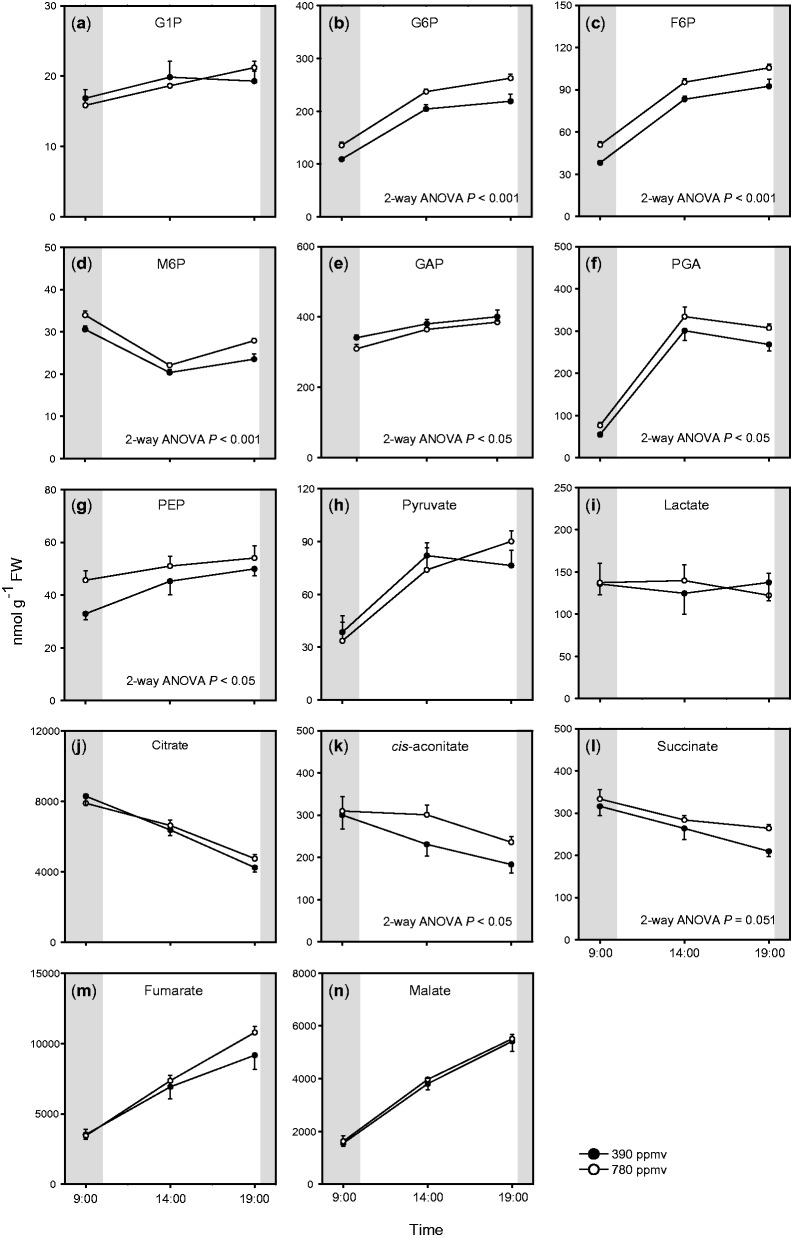
Diurnal changes in levels of sugar phosphates and organic acids in *A. thaliana* shoots. Mean values ± SEM are shown (*n* = 4–5). For other details, see the legend of Fig. 1.

**Fig. 6:**
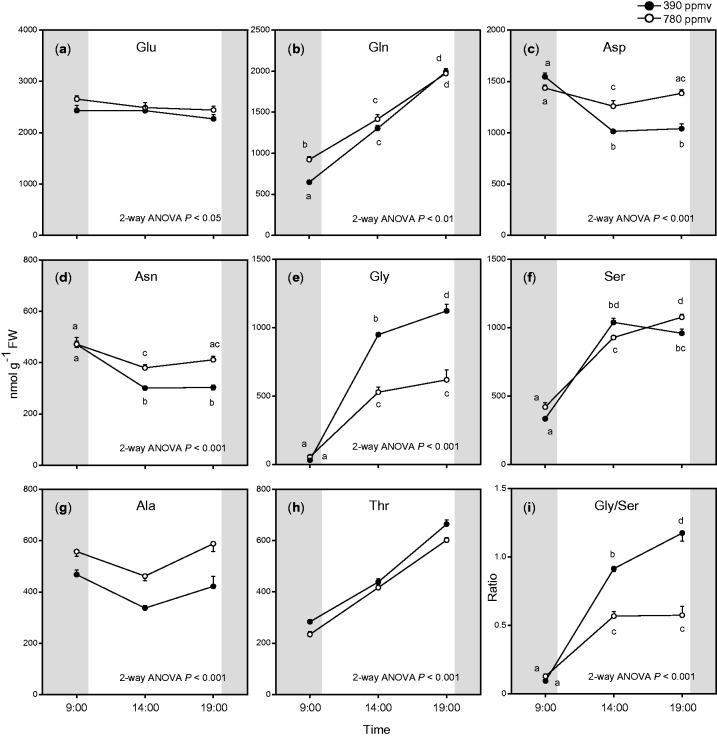
Diurnal changes in levels of major amino acids in *A. thaliana* shoots. Mean values ± SEM are shown (*n* = 5). For other details, see the legend of Fig. 1.

We measured levels of primary metabolites including hexose phosphates (hexose-P), organic acids and amino acids in the shoots. There were substantial diurnal changes in the levels of most metabolites, irrespective of the CO_2_ level (Figs. 5, 6; Supplementary Fig. S3). We quantified four hexose-P: glucose-1-phosphate (G1P), glucose-6-phosphate (G6P), fructose-6-phosphate (F6P) and mannose-6-phosphate (M6P) (Fig. 5a–d). The levels of G6P and F6P were higher than those of G1P and M6P. G6P and F6P accumulated during the day and their levels were higher under elevated CO_2_ than under moderate CO_2_. We also quantified glyceraldehyde-3-phosphate (GAP), 3-phosphoglycerate (PGA), phospho*enol*pyruvate (PEP) and pyruvate, which are intermediates of glycolysis (Fig. 5e–h). PGA and PEP showed diurnal patterns similar to that of G6P, and the levels of these metabolites were significantly higher under elevated CO_2_ than under moderate CO_2_. In contrast, there were lower levels of GAP under elevated CO_2_ than under moderate CO_2_, but there were only small differences in GAP and pyruvate levels between the two CO_2_ conditions. The total amounts of hexose-P (G1P, G6P, F6P and M6P) and glycolysis intermediates (Fig. 4b, c) increased during the day, and the effects of elevated CO_2_ on their levels were most prominent at the end of the light period. These results indicate that the levels of some intermediates of glycolysis increased with increasing starch levels in shoots of Arabidopsis grown under elevated CO_2_.

The total amount of organic acids in the TCA cycle also increased at the end of the light period under elevated CO_2_ (Fig. 4d). The diurnal patterns of intermediates of the TCA cycle were divided into two groups (Fig. 5j–n); one group showed decreased levels during the day (citrate, *cis*-aconitate and succinate) while the other showed increased levels during the day (fumarate and malate). There were large differences in the levels of TCA cycle intermediates between the two CO_2_ conditions at the end of the light period. The levels of *cis*-aconitate and succinate were higher under elevated CO_2_ than under moderate CO_2_.

Amino acids are synthesized from intermediates of glycolysis or the TCA cycle. We measured levels of 20 amino acids (Fig. 6; Supplementary Fig. S3), and found that the total amount of amino acids was significantly enhanced under elevated CO_2_ (Fig. 4e). The levels of several major amino acids (glutamate, aspartate, asparagine and alanine) were significantly higher under elevated CO_2_ than under moderate CO_2_, especially in the middle of the day and at the end of the light period (Fig. 6). In contrast, glycine, an intermediate of photorespiration, accumulated during the day but showed a lower level under elevated CO_2_ than under moderate CO_2_ (Fig. 6e). The ratio of glycine to serine was lower under elevated CO_2_ (Fig. 6i) than under moderate CO_2_. This may be because of suppressed photorespiration under elevated CO_2_. The levels of some other minor amino acids showed small differences between the two CO_2_ conditions (Supplementary Fig. S3).

**Fig. 7:**
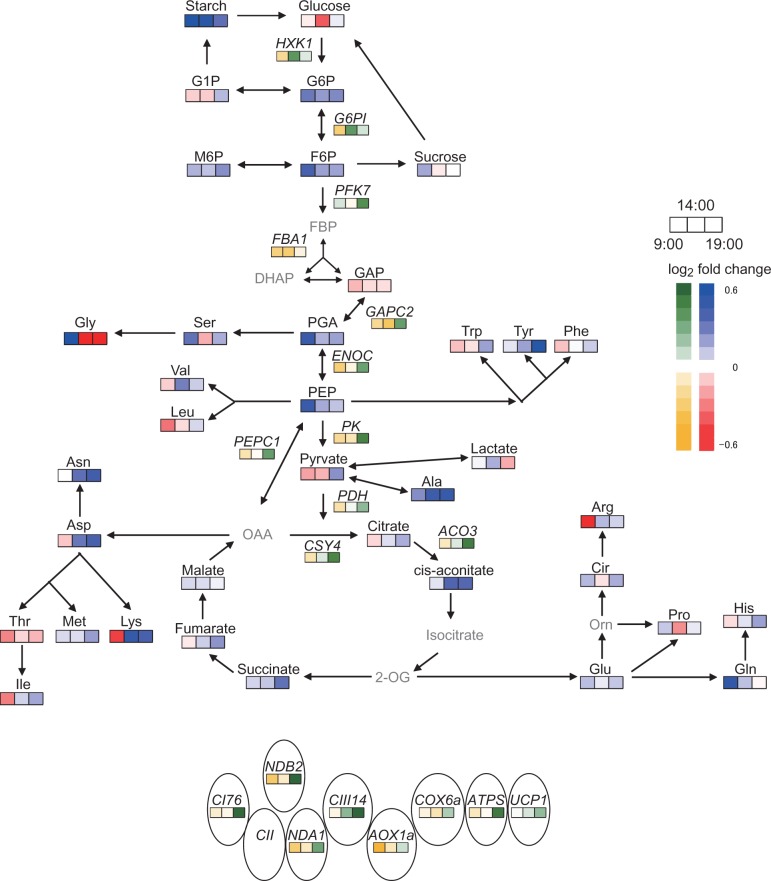
Heat map of differences in levels of metabolites and transcripts in the respiratory system between moderate and elevated CO_2_. The ratio of the value under elevated CO_2_ to that under moderate CO_2_ was calculated, and then the log_2_-fold change in this ratio was plotted. Blue or red indicate log_2_-fold changes in levels of primary metabolites, and green or yellow indicate log_2_-fold changes in transcript levels. Gray letters denote that the metabolites could not be detected or were not measured.

For each transcript or metabolite, we calculated the ratio of the value under elevated CO_2_ to that under moderate CO_2_ using transcript data (Figs. 1, 2) or primary metabolite data (Figs. 4–6; Supplementary Figs. S2, S3). The log_2_-fold change in these ratios is shown in a metabolic map (Fig. 7). The levels of starch, hexose-P and some respiratory intermediates increased under elevated CO_2_, especially at the end of the light period. The levels of some amino acids increased under elevated CO_2_, but no specific pathway of amino acid synthesis was activated under elevated CO_2_.

### Response of respiratory rates to elevated CO_2_

**Fig. 8:**
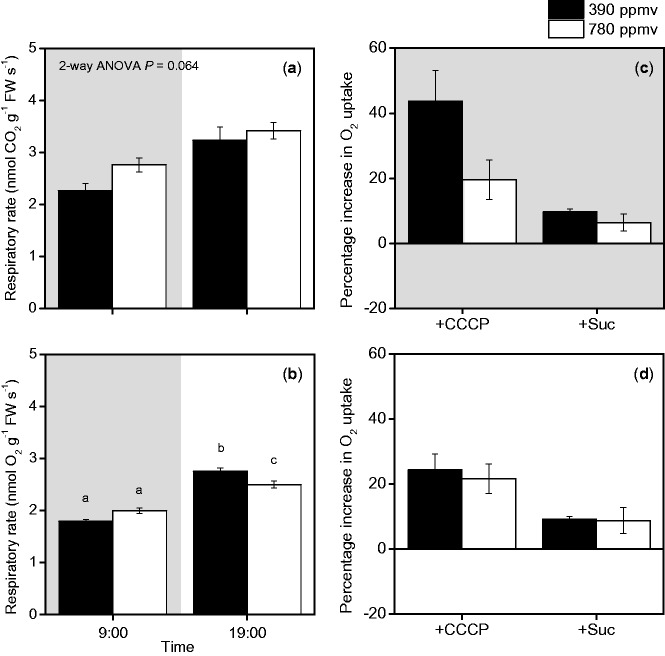
Diurnal changes in rates of CO_2_ efflux (a) and O_2_ uptake (b) in *A. thaliana* shoots. Percentage increase in O_2_ uptake rates after addition of CCCP, an uncoupler (+CCCP), and sucrose (+Suc) to shoots at the end of the dark period (c) and at the end of the light period (d). Black and white bars represent plants grown under moderate or elevated CO_2_, respectively. Mean values ± SEM are shown (*n* = 4–12). For other details, see the legend of Fig. 1.

Although the maximal activities of enzymes were similar in the two CO_2_ treatments (Fig. 3), there were higher levels of starch and intermediates of glycolysis under elevated CO_2_ than under moderate CO_2_ (Figs. 4, 5, 7). We expected that the increased levels of these metabolites would affect the respiratory rate because of the increase in substrate availability. Changes in the respiratory rate could also result from changes in the ATP consumption rate, which may increase as a result of enhanced plant growth (Supplementary Fig. S1; Supplementary Tables S1, S2), and/or increased carbohydrate export from the leaves associated with carbohydrate accumulation (Fig. 4). We measured the rates of CO_2_ efflux and O_2_ uptake in shoots at the ends of the day and night periods. The CO_2_ efflux and O_2_ uptake rates increased during the day. At the end of the night period, the rates of CO_2_ efflux and O_2_ uptake under elevated CO_2_ were higher than those under moderate CO_2_ (Fig. 8a, b). At the end of the light period, the rate of O_2_ uptake under elevated CO_2_ was lower than that under moderate CO_2_ (Fig. 8b).

To determine whether the O_2_ uptake rate was limited by the availability of respiratory substrates or the ATP consumption rate, we examined the effects of adding sucrose or an uncoupler, carbonyl cyanide *m*-chlorophenyl hydrazine (CCCP), on the O_2_ uptake rate (Fig. 8c, d). The addition of sucrose increased the O_2_ uptake rate by <10% in the shoots of plants under both CO_2_ levels. The addition of CCCP increased the O_2_ uptake rates more than did the addition of sucrose, particularly in the shoots of plants grown under moderate CO_2_ at the end of the night period. These results indicate that the O_2_ uptake rate was primarily limited by the ATP consumption rate in *A. thaliana* shoots. Therefore, the difference in the ATP consumption rate explained the difference in the O_2_ uptake rates between plants under elevated and moderate CO_2_ conditions (Fig. 8b).

After the addition of both CCCP and sucrose, the O_2_ uptake rate in shoots under elevated CO_2_ (2.61 ± 0.04 µmol O_2 _g^−1 ^FW s^−1^, mean ± SEM) was lower than that in shoots under moderate CO_2_ (2.89 ± 0.12 µmol O_2_ g^−1 ^FW s^−1^) at the end of the night period. A similar pattern was observed at the end of the light period (3.24 ± 0.12 µmol O_2_ g^−1 ^FW s^−1^ in shoots under elevated CO_2_ and 3.62 ± 0.09 µmol O_2_ g^−1 ^FW s^−1^ in shoots under moderate CO_2_). These results indicate that the respiratory capacity of plants was higher under moderate CO_2_ than under elevated CO_2_, although there was no difference in the maximal enzyme activities between the two CO_2_ conditions.

## Discussion

In this study, we examined the responses of the respiratory system to elevated CO_2_ in *A. thaliana* shoots. Our main findings were as follows. (i) At the end of the light period, the transcript levels of respiratory enzymes were higher under elevated CO_2_ than under moderate CO_2_. However, these increases did not correspond to increased maximal activities of the corresponding respiratory enzymes. (ii) The levels of several primary metabolites were higher under elevated CO_2_ than under moderate CO_2_, especially at the end of the light period, but these increases in primary metabolite levels did not enhance the respiratory rate under elevated CO_2_, even at the end of the light period. (iii) The O_2_ uptake rate was determined mainly by the consumption rate of respiratory ATP in *A. thaliana* shoots. Elevated CO_2_ may affect anabolic processes that consume respiratory ATP, thereby affecting the O_2_ uptake rate.

### Changes in transcript levels and enzyme activities under elevated CO_2_

The transcript levels of genes encoding respiratory enzymes were higher under elevated CO_2_ (Figs. 1, 2, 7) than under moderate CO_2_. The up-regulation of *ENOC* and *PEPC1* under elevated CO_2_ (Figs. 1, 7) is consistent with the results of other studies (Li et al. 2008, Leakey et al. 2009b). Li et al. (2008) reported that transcript levels of *PEPC1* were increased in *A. thaliana* shoots at 7 d after transfer to elevated CO_2_ conditions. Leakey et al. (2009b) also observed that the expression levels of enolase and PEPC were enhanced in soybean leaves under elevated CO_2_. In the present study, the transcript levels not only of *PEPC1* but also those of *PDH*, *CSY4* and *ACO3* were up-regulated at the end of the light period under elevated CO_2_. These increases may reflect increased fluxes of anaplerotic or non-cyclic TCA pathways, which would lead to increased levels of TCA cycle intermediates and amino acids. The transcript levels of *CI76*, *CIII14* and *ATPS*, which encode components of respiratory chain complexes related to respiratory ATP production, were enhanced at the end of the light period under elevated CO_2_ (Figs. 2, 7). The diurnal patterns of these transcripts were similar to those of several genes encoding enzymes involved in glycolysis and the TCA cycle, which also showed increased transcript levels under elevated CO_2_. We analyzed the promoter regions of these genes using database tools, but we did not find any *trans*- or *cis*-acting factors responsible for their up-regulation. Further studies are needed to elucidate this coordinated pattern of up-regulation. On the other hand, genes encoding enzymes related to non-phosphorylating pathways did not show clear responses to elevated CO_2_ (Fig. 2e–h). Previous studies (Li et al. 2006, Li et al. 2008) reported similar results.

Although the number of mitochondria often increases under elevated CO_2_ (Griffin et al. 2001, Wang et al. 2004), it was reported that maximal COX activity was suppressed under elevated CO_2_ in several plant species (Azcon-Bieto et al. 1994, Gonzalez-Meler et al. 1996; Gomez-Casanovas et al. 2007). The in vivo flux via the cytochrome pathway was also suppressed under elevated CO_2_ (Gonzalez-Meler et al. 2004, Gonzalez-Meler and Taneva 2005, Gomez-Casanovas et al. 2007). In contrast, the in vivo flux via AOX was increased under elevated CO_2_ (Gonzalez-Meler et al. 2004). In this study, we did not examine the in vivo flux via AOX and its redox state, although our results showed that the transcript level of *AOX1a* was not up-regulated under elevated CO_2_ (Fig. 2). Our preliminary results indicated that the *aox1a* mutant showed a phenotype similar to that of wild-type *A. thaliana* under elevated CO_2_. However, Gandin et al. (2012) reported that the CO_2_ assimilation rate and quantum efficiency of PSII were lower in the *aox1a* mutant than in the wild type under elevated CO_2_. Since they cultivated the plants only under ambient CO_2_ conditions, it is still unclear whether AOX has important roles in growth and photosynthesis under elevated CO_2_. However, AOX has an important role in the interaction between photosynthesis and respiration (Noguchi and Yoshida 2008), and changes in the contribution of AOX greatly affect respiratory ATP production. Further research should clarify the roles of AOX in growth and photosynthesis under elevated CO_2_.

Sweetlove et al. (2006) demonstrated that the rates of photorespiratory glycine oxidation and CO_2_ assimilation were lower in the *ucp1* mutant than in the wild type. We did not analyze the function of UCP1 under elevated CO_2_, but our results showed that the transcript level of *UCP1* was marginally increased at the end of the light period under elevated CO_2_ (Fig. 2h). Since UCP also affects respiratory ATP production, further research is required to clarify the functions of this enzyme under elevated CO_2_.

### Responses of primary metabolites to elevated CO_2_

Our results showed that under elevated CO_2_, the levels of the glycolysis intermediates hexose-P, PGA and PEP were increased in *A. thaliana* shoots. Arrivault et al. (2009) reported that the levels of hexose-P and some other intermediates of glycolysis were enhanced in *A. thaliana* shoots under elevated CO_2_, although the plants were exposed to only a 15 min elevated CO_2_ treatment in their experiments. Li et al. (2008) also reported enhanced levels of carbohydrates in two ecotypes of *A. thaliana* under elevated CO_2_. In shoots, higher levels of CO_2_ enhanced photosynthetic C gain, which increased the levels of carbohydrates and glycolysis intermediates.

Disruption of photorespiration was reported to affect the amino acid composition in plant tissues (e.g. Igarashi et al. 2006). The suppression of photorespiration under elevated CO_2_ may affect the amino acid composition, but, in this study, the total amounts of amino acids were higher under elevated CO_2_ than under moderate CO_2_ (Fig. 4). Synthesis of some amino acids may be up-regulated by increased levels of some glycolysis intermediates (Fig. 7). In this study, alanine accumulated during the day under elevated CO_2_, whereas pyruvate, a precursor of alanine, did not increase under elevated CO_2_ (Figs. 5–7). In *Lotus japonicas* roots, alanine accumulated under hypoxia, but pyruvate did not (Rocha et al. 2010). The accumulation of alanine under elevated CO_2_ may result from the up-regulation of alanine aminotransferase.

There were two main patterns of diurnal changes in the levels of TCA cycle intermediates; citrate, *cis*-aconitate and succinate accumulated during the night, while fumarate and malate accumulated during the day (Fig. 5). These patterns were similar to those reported in a previous study (Gibon et al. 2006). In illuminated leaves, pyruvate dehydrogenase is suppressed (Tovar-Mendez et al. 2003). This may result in partial operation of the TCA cycle (Sweetlove et al. 2010), leading to the accumulation of malate and fumarate. These organic acids may be consumed as respiratory substrates during the night. On the other hand, the accumulated citrate may provide a source of C skeletons for nitrogen (N) assimilation during the day (Gauthier et al. 2010). The accumulation of citrate may have increased N assimilation, leading to the increase in the total amount of amino acids during the day (Fig. 4e).

### Response of respiratory rate to elevated CO_2_

The rates of both CO_2_ efflux and O_2_ uptake decreased during the night in *A. thaliana* shoots (Fig. 8). These decreases were similar to those reported in previous studies (Noguchi et al. 1996, Noguchi and Terashima 1997). The O_2_ uptake rate is generally limited by substrate availability, enzyme activity or the ATP consumption rate. Based on the data in Fig. 3, enzyme capacity would not limit the O_2_ uptake rate in *A. thaliana* shoots on day 20. At the end of the light period, the levels of starch and intermediates of glycolysis and the TCA cycle in shoots were higher under elevated CO_2_ than under moderate CO_2_. If the O_2_ uptake rate is determined by respiratory substrate availability, then the O_2_ uptake rate should be higher under elevated CO_2_ than under moderate CO_2_ at the end of the light period; however, we found that the O_2_ uptake rate was higher under moderate CO_2_ than under elevated CO_2_ at the end of the light period (Fig. 8b). This finding suggested that substrate availability did not directly affect the O_2_ uptake rate in *A. thaliana* shoots in these conditions. The results of the experiments in which substrate or uncoupler were added supported this idea. The addition of sucrose only slightly increased the O_2_ uptake rate (Fig. 8c, d), while addition of the uncoupler strongly increased the O_2_ uptake rate, especially at the end of the night period (Fig. 8c, d).

Many physiological processes that consume the respiratory ATP, such as growth, N assimilation and carbohydrate export, were affected by the CO_2_ conditions. Based on the rates of CO_2_ efflux and the decrease in starch during the night, we estimated the rate of carbohydrate export from the shoots and the cost of carbohydrate export using calculations described previously (Noguchi et al. 2001). We assumed that a portion of the starch stored during the day was consumed by respiration, and the remainder was converted to sucrose and apoplastically exported from the leaves. The rate of carbohydrate export, and, therefore, the cost of carbohydrate export, were higher under elevated CO_2_ than under moderate CO_2_ (1.47 nmol ATP g^−1 ^FW s^−1^ under elevated CO_2_ and 1.11 nmol ATP g^−1 ^FW s^−1^ under moderate CO_2_). Given that the ATP/O_2_ ratio of respiratory ATP production is 29/6, the cost was estimated at 8.3% and 15.3% of the ATP produced by respiration during the night under moderate and elevated CO_2_, respectively.

The CO_2_ efflux rate was higher under elevated CO_2_ than under moderate CO_2_ at the ends of the light and dark periods (Fig. 8a). Why did the responses of the CO_2_ efflux rate and the O_2_ uptake rate differ? The ratio of the CO_2_ efflux rate to O_2_ uptake rate was higher under elevated CO_2_ than under moderate CO_2_ in both light and dark periods (data not shown), although we could not calculate exact RQ values because we used different measurement systems. An increase in the ratio indicates consumption of organic acids as respiratory substrates. The organic acids in the TCA cycle accumulated in *A. thaliana* shoots under elevated CO_2_ (Fig. 4d). Consistent with this, a higher RQ value was observed in soybean leaves under elevated CO_2_ (Leakey et al. 2009b). Anabolic processes such as nitrate assimilation were also enhanced under elevated CO_2_; these would contribute to the high RQ, as suggested by the flux-balanced model (Buckley and Adams 2011). Further research should focus on clarifying which anabolic processes are stimulated in shoots under elevated CO_2_.

In this study, our results showed that elevated CO_2_ affected ATP-consuming processes, thereby affecting the rate of O_2_ uptake. Our data also indicated that the rates of respiratory CO_2_ efflux may depend on NADH consumption by the respiratory chain and by various anabolic processes in the growing shoots of *A. thaliana*. The increase in atmospheric CO_2_ is expected to lead to increases in temperature, which will strongly affect the respiratory system of plants (Atkin and Tjoelker 2003). Ayub et al. (2011) showed that the growth temperature affected the responses of leaf respiratory rates to elevated CO_2_. A meta-analysis indicated that the responses of some metabolites to elevated CO_2_ were mitigated by a temperature increase (Zvereva and Kozlov 2006). We should conduct further detailed analyses on respiratory responses to increased temperatures under elevated CO_2_.

When considering the respiratory responses of terrestrial plants to elevated CO_2_, the responses of stems and roots should be taken into account. Under the same conditions as those used in this study, the growth and respiratory O_2_ uptake rates of whole roots in *A. thaliana* were increased under elevated CO_2_ (data not shown). Körner et al. (2005) analyzed changes in leaf properties and stem growth in 35 m tall temperate forest trees subjected to elevated CO_2_. They found that one *Fagus* species showed a transient increase in stem growth, but the responses differed among species. Organic exudation from roots was reported to increase under elevated CO_2_ (Allard et al. 2005, Phillips et al. 2006). The increase in photosynthetically fixed C under elevated CO_2_ does not always result in increased plant growth. The responses of stem and root respiration and/or exudation from roots may also be important in the overall plant response to elevated CO_2_.

There were no other stresses in the growth conditions in this study. Therefore, the *A. thaliana* plants may have shown optimal responses to elevated CO_2_. However, it is important to note that stress conditions would affect the responses of respiratory systems to elevated CO_2_. Ayub et al. (2011) showed that the responses of leaf respiratory rates were altered by a drought treatment. Also, we cannot necessarily extrapolate these results to other species. It is important to evaluate the respiratory responses to elevated CO_2_ in species that show a large ratio of respiratory consumption to gross photosynthetic production; for example, evergreen tree species. The respiratory responses of these species will greatly affect the C balance in terrestrial ecosystems in the future.

## Materials and Methods

### Plant materials and growth conditions

*Arabidopsis thaliana* (L.) Heynh. accession Columbia was used in these experiments. Seeds were sown on plates of modified MGRL medium (MGRL-base medium), consisting of 10 mM KNO_3_, 2 mM CaCl_2_, 1.5 mM NaH_2_PO_4_, 1.5 mM MgSO_4_, 0.26 mM Na_2_HPO_4_, 30 µM H_3_BO_3_, 12 µM Fe(III)·EDTA·3H_2_O, 10 µM MnSO_4_·5H_2_O, 1.0 µM ZnSO_4_·7H_2_O, 0.96 µM CuSO_4_·5H_2_O, 126 nM CoCl_2_·6H_2_O, 24 nM (NH_4_)_6_ Mo_7_O_24_4H_2_O and 0.25% (w/v) gellan gum. The medium did not contain sucrose. The pH of the medium was adjusted to 6.0 with KOH. Fifty seeds were sown on each plate (8.5 cm in diameter) containing 30 ml of MGRL-base medium. After sowing, the plates were kept at 4°C in the dark under ambient CO_2_ conditions for 2 d, and then transferred to CO_2_-controlled growth chambers (LPH-0.5P-SH; Nippon Medical & Chemical Instrument) at 23°C and 60% relative humidity. The CO_2_ concentration in the chambers was controlled at 390 or 780 ppmv CO_2_ (moderate or elevated CO_2_, respectively). The day length was 10 h (from 10:00 to 20:00 h), and the photosynthetically active photon flux density (PPFD) was 100–130 µmol m^−2 ^s^−1^. Seeds were germinated under moderate or elevated CO_2_. At day 7, the seedlings were transferred to new plates containing 40 ml of MGRL-base medium. The density was reduced to two seedlings per plate, which was sufficiently low to avoid N deficiency for plant growth. Other growth conditions were the same as described above. The positions of the plates were periodically changed, and we changed the CO_2_ concentrations in the growth chambers as well (i.e. elevated and moderate CO_2_ conditions were not always applied in the same chamber). We conducted the following analyses and samplings using plants cultivated in more than two independent experiments.

### Growth analysis

To analyze RGR, plants were harvested every other day from day 10 to day 30, between 14:00 and 16:00 h. Plants were cut into shoots and roots, and whole shoots were used for measurements. These samples were harvested, weighed, and dried at 80°C for 3 d. RGR was calculated as the slope of a linear regression of the natural logarithm of plant mass as a function of time using the data of three sequential samplings.

### Sampling on day 20

To analyze the diurnal changes in the respiratory rate, plants were harvested at the end of the night period (from 09:00 to 10:00 h) and at the end of the light period (from 19:00 to 20:00 h) on day 20. To analyze the diurnal changes in primary metabolites, transcript levels and maximal activities of respiratory enzymes, plants were harvested at the end of the night period (from 09:00 to 10:00 h), in the middle of the day (from 14:00 to 15:00 h) and at the end of the light period (from 19:00 to 20:00 h) on day 20. Plants were cut into shoots and roots, and whole shoots were used for measurements. The samples were rapidly harvested at each time point, weighed and immediately frozen in liquid N.

### Extraction of total RNA and reverse transcription–PCR (RT–PCR)

Total RNA was extracted from frozen shoots using TRIzol Reagent (Life Technologies Corp.) according to the manufacturer’s instructions. We used a TURBO DNA-free Kit (Life Technologies Corp.) to remove DNA from RNA preparations. RT–PCR was performed using the extracted RNA and a High Capacity RNA-to-cDNA Kit (Life Technologies Corp.).

### Real-time PCR

Transcript levels were measured using a 7300 Real-time PCR System (Life Technologies Corp.). The reaction mixtures contained 1 µl of cDNA, 12.5 µl of 2× Power SYBR Green PCR Master Mix (Life Technologies Corp.), 0.5 µl of specific primer (final concentration, 0.2 µM) and 10.5 µl of sterilized water. The PCR conditions were as follows: 50°C for 2 min, 95°C for 10 min and 40 cycles of 95°C for 15 s followed by 60°C for 1 min. Relative transcript levels were calculated by the comparative cycle threshold method. After examining the most suitable internal standard gene among 40S ribosomal protein S15A (*RPS15aA*; At1g07770), elongation factor 1-α (*EF1-α*; At5g60390), actin 3 (*ACT3*; At3g53750), ubiquitin 10 (*UBQ10*; At4g05320) and 18S rRNA (*18S rRNA*; At2g01010), we selected *RPS15aA* as the internal standard. The primer sequences are shown in Supplementary Table S3.

### Measurements of maximal activities of respiratory enzymes

Frozen shoots were ground in liquid N and extracted in buffer [50 mM KH_2_PO_4_–K_2_HPO_4_ (pH 7.6), 10 mM MgSO_4_, 1 mM EDTA, 5 mM dithiothreitol (DTT), 0.05% (v/v) Triton X-100, one tablet of protease inhibitor cocktail (Rochey) per 30 ml of buffer and 5% (w/v) insoluble polyvinylpyrrolidone]. The mixture was centrifuged at 10,000×*g* at 4°C for 3 min, and the upper aqueous phase was used for measurements of enzyme activities. The maximal activities of enolase, PEPC and fumarase were determined as described in Lance and Guy (1992), Fukayama et al. (2003) and Cooper and Beevers (1969), respectively. The maximal activities of citrate synthase and aconitase were determined as described by MacDougall and Rees (1991).

### Measurement of non-structural carbohydrate content

Non-structural carbohydrates (starch, glucose and sucrose) were quantified as described by Watanabe et al. (2008), with slight modifications. Frozen shoots were ground with a Multi-Beads Shocker (Yasui Kikai) using metal cones at 2,000 r.p.m. for 10 s. After adding 1 ml of 80% ethanol, the suspension was incubated at 80°C for 10 min, and then centrifuged at 1,500×*g* at 4°C for 10 min. The precipitate was used for determination of starch. Ethanol was removed from the supernatant by evaporation using a centrifugal concentrator (CC-105; Tomy). Equal volumes of distilled water and chloroform were added to the concentrated supernatant, the mixture was mixed well, and then centrifuged at 10,000×*g* at 4°C for 10 min. The upper aqueous phase was used for determination of glucose and sucrose. The precipitate was suspended in distilled water and boiled for 60 min at 100°C. An equal volume of amyloglucosidase was added to the boiled suspension and incubated for 60 min at 55°C. The mixture was centrifuged at 10,000×*g* at 4°C for 10 min, and the upper aqueous phase was used for determination of starch. A glucose-C2-test (Wako) was used for detection.

### Determination of metabolites by capillary electrophoresis-mass spectrometry (CE-MS)

Metabolites were extracted and determined according to the method of Sato et al. (2004) and Sato and Yanagisawa (2010), with slight modifications. Frozen shoots were ground with a Multi-Beads Shocker (Yasui Kikai) using metal cones at 2,000 r.p.m. for 10 s. After adding 200 µl of ice-cold 80% (v/v) methanol, an equal volume of an internal standard solution containing 200 µM PIPES and l-methionine sulfone was added to the extract, mixed well and then the mixture was centrifuged at 2,000×*g* for 1 min at 4°C. The supernatant was added to a centrifugal concentrator with a 3 kDa cut-off membrane (PALL Corporation) and centrifuged at 15,000×*g* for 15 min at 4°C. The filtered samples were freeze-dried using an Alpha 2-4 LDplus Freeze Dryer (Martin Christ) and stored at −80°C. The samples were solved in Milli Q water and then analyzed using a capillary electrophoresis system with a built-in diode-array detector, an 1100 series MSD mass spectrometer, an 1100 series isocratic HPLC pump, a G1603A CE-MS adapter kit and a G1607A CEESI-MS sprayer kit (all Agilent Technologies). The amounts of metabolites were corrected against those of internal standards.

### Measurements of respiratory rates

Respiratory CO_2_ efflux rates of shoots were measured using a laboratory-constructed system as described by Tazoe et al. (2008), with some modifications. The leaf chamber was a 0.3 liter, 150 mm × 100 mm × 20 mm aluminum box with a glass window. The chamber was equipped with two small fans to circulate air, and two copper–constantan thermocouples to monitor leaf temperature. The air temperature inside the chamber was regulated by circulating water in a jacket attached to the chamber, and the leaf temperature was kept at 23°C. The flow rate of air entering the chamber, monitored with a mass-flow meter, was controlled at 300 ml min^−1^. Partial pressures of H_2_O and CO_2_ in the gas were measured with an infrared gas analyzer (LI-7000, Li-Cor). The CO_2_ partial pressure in the air entering the leaf chamber was maintained at 39 or 78 Pa for the shoots grown under moderate or elevated CO_2_, respectively. Gas exchange parameters were calculated according to von Caemmerer and Farquhar (1981). Before measurement, plants in plates were kept in the dark for 20 min. The shoot was separated from the root and inserted into a lump of a small piece of the gellan gum medium to prevent dehydration during measurement. Four shoots from the moderate CO_2_ treatment or two shoots from the elevated CO_2_ treatment were used for each measurement. After measurement, the shoots were dried at 80°C for 3 d and weighed. To express values on a FW basis, we used the ratio of DW to FW, which was determined using separate plants grown at the same time as those used for gas exchange measurements. The ratios used were 0.0569 for the moderate CO_2_ treatment and 0.0585 for the elevated CO_2_ treatment at the end of the night period, and 0.0673 for the moderate CO_2_ treatment and 0.0667 for the elevated CO_2_ treatment at the end of the light period.

The respiratory O_2_ uptake rate of shoots was measured using a liquid-phase Clark-type O_2_ electrode (Rank Brothers) at 23°C. Before measuring respiratory rates, the shoots were incubated in buffer [50 mM HEPES, 10 mM MES (pH 6.6) and 0.2 mM CaCl_2_] for 20 min in the dark. The O_2_ uptake rate was calculated assuming that the concentration of O_2_ in the air-saturated buffer was 260 µM at 23°C. After a constant rate of O_2_ uptake was attained in the buffer without effectors, sucrose (final concentration, 100 mM) or CCCP (final concentration, 10 µM) was added and the changes in the O_2_ uptake rate were analyzed. Preliminary experiments had shown that these concentrations were the most effective.

### Statistical analyses

All statistical analyses were conducted with Microsoft Excel 2010 (Microsoft) and SPSS 12.0 J (SPSS). The results of two-way analysis of variance (ANOVA) are shown in Supplemenatry Table S4.

## Supplementary data

Supplementary data are available at PCP online.

## Funding

This work was supported by the Ministry of Education, Culture, Sports, Science and Technology, Japan [Grants-in-Aid for Scientific Research on Innovative Areas (21114007)].

## Supplementary Material

Supplementary Data

## Abbreviations

ANOVA: analysis of variance
CCCP: carbonyl cyanide *m*-chlorophenyl hydrazine
CE-MS: capillary electrophoresis–mass spectrometry
COX: cytochrome *c* oxidase
F6P: fructose-6-phosphate
G1P: glucose-1-phosphate
G6P: glucose-6-phosphate
GAP: glyceraldehyde-3-phosphate
hexose-P: hexose phosphates
LMA: leaf mass per area
M6P: mannose-6-phosphate
PEP: phospho*enol*pyruvate
PGA: 3-phosphoglycerate
RGR: relative growth rate
RQ: respiratory quotient
RT–PCR: reverse transcription–PCR
TCA: tricarboxylic acid
