# Supplementary material for: Effects of Elevated CO2 on Levels of Primary Metabolites and Transcripts of Genes Encoding Respiratory Enzymes and Their Diurnal Patterns in Arabidopsis thaliana: Possible Relationships with Respiratory Rates
Source: Plant Cell Physiol. 2014 Jan 18;55(2):341–57. doi: 10.1093/pcp/pct185 (PMC3913440; doi:10.1093/pcp/pct185)
Supplement: Supplementary Data [file supp_55_2_341__index.html]

Effects of Elevated CO2 on Levels of Primary Metabolites and Transcripts of Genes Encoding Respiratory Enzymes and Their Diurnal Patterns in Arabidopsis thaliana: Possible Relationships with Respiratory Rates — Effects of Elevated CO2 on Levels of Primary Metabolites and Transcripts of Genes Encoding Respiratory Enzymes and Their Diurnal Patterns in Arabidopsis thaliana: Possible Relationships with Respiratory Rates — Effects of Elevated CO2 on Levels of Primary Metabolites and Transcripts of Genes Encoding Respiratory Enzymes and Their Diurnal Patterns in Arabidopsis thaliana: Possible Relationships with Respiratory Rates — Supplementary Data 

# Effects of Elevated CO2 on Levels of Primary Metabolites and Transcripts of Genes Encoding Respiratory Enzymes and Their Diurnal Patterns in *Arabidopsis thaliana*: Possible Relationships with Respiratory Rates

## Supplementary Data

files

**Files in this Data Supplement:**

- Supplementary Data - pdf file
- Supplementary Data - pdf file
- Supplementary Data - pdf file
- Supplementary Data - docx file
- Supplementary Data - docx file
- Supplementary Data - doc file
- Supplementary Data - doc file
