# Supplementary material for: Effects of Elevated CO2 on Levels of Primary Metabolites and Transcripts of Genes Encoding Respiratory Enzymes and Their Diurnal Patterns in Arabidopsis thaliana: Possible Relationships with Respiratory Rates
Source: Plant Cell Physiol. 2014 Jan 18;55(2):341–57. doi: 10.1093/pcp/pct185 (PMC3913440; doi:10.1093/pcp/pct185)
Supplement: Supplementary Data [file supp_pct185_pcp-2013-e-00454-File010.pdf]

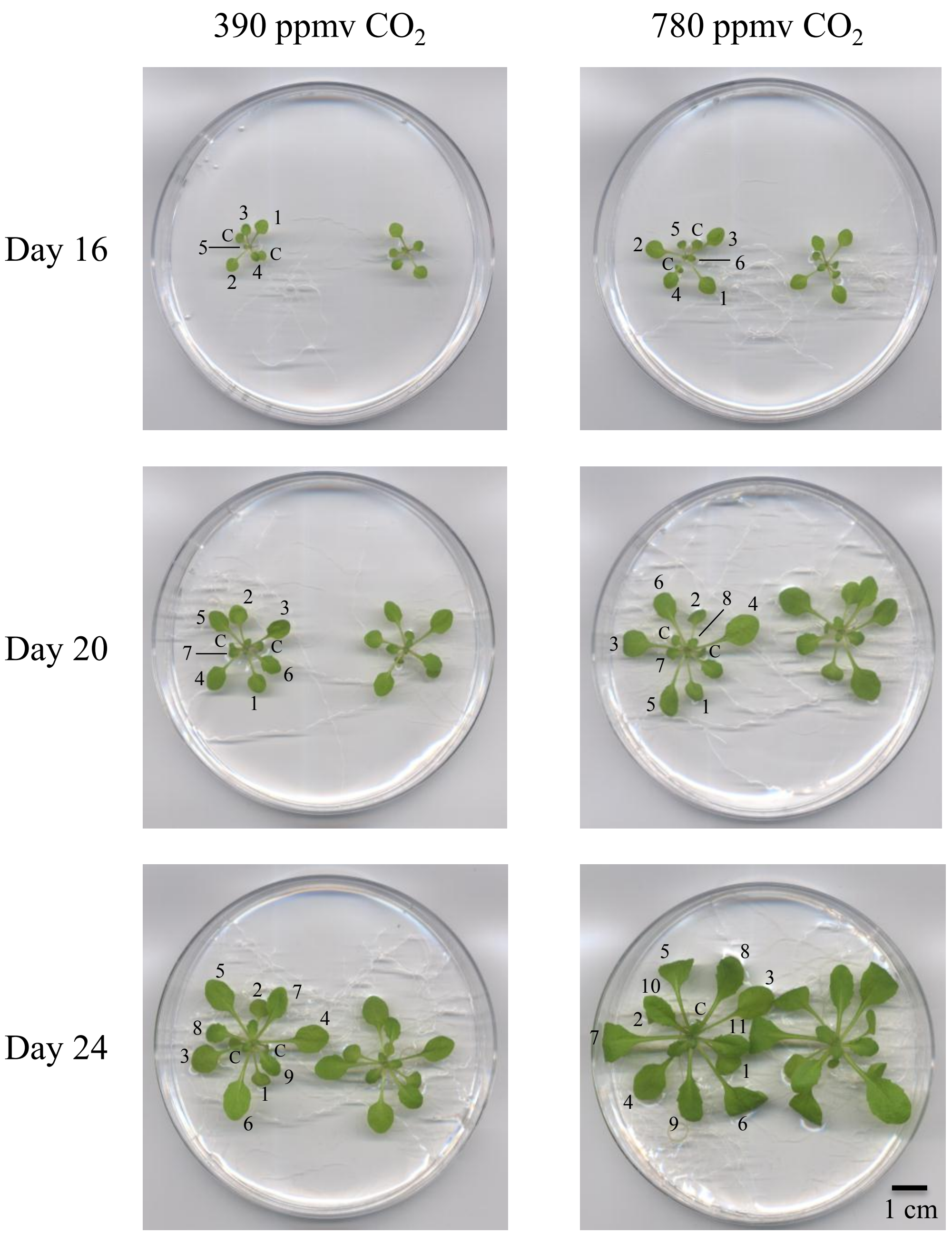

Figure S1: Day-16, 20 and 24 plants grown at 390 or 780 ppmv CO2. The numbers denote the leaf order, and C indicates the cotyledon. In the elevated CO2, plants grew faster and leaf numbers increased more than in the ambient CO2. Scale bar shows 1 cm.
