# Supplementary material for: Effects of Elevated CO2 on Levels of Primary Metabolites and Transcripts of Genes Encoding Respiratory Enzymes and Their Diurnal Patterns in Arabidopsis thaliana: Possible Relationships with Respiratory Rates
Source: Plant Cell Physiol. 2014 Jan 18;55(2):341–57. doi: 10.1093/pcp/pct185 (PMC3913440; doi:10.1093/pcp/pct185)
Supplement: Supplementary Data [file supp_pct185_pcp-2013-e-00454-File011.pdf]

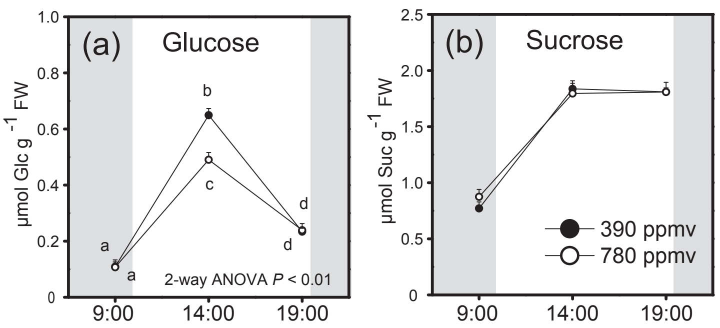

Figure S2: Diurnal changes in levels of sucrose and glucose in A. thaliana shoots. Mean ± SEM are shown (n = 4-5). For other details, see the legend of Figure 1.
