# Supplementary material for: Effects of Elevated CO2 on Levels of Primary Metabolites and Transcripts of Genes Encoding Respiratory Enzymes and Their Diurnal Patterns in Arabidopsis thaliana: Possible Relationships with Respiratory Rates
Source: Plant Cell Physiol. 2014 Jan 18;55(2):341–57. doi: 10.1093/pcp/pct185 (PMC3913440; doi:10.1093/pcp/pct185)
Supplement: Supplementary Data [file supp_pct185_pcp-2013-e-00454-File013.docx]

Table S1. Dry weight (DW) of shoots of *A. thaliana* grown at 390 and 780 ppmv CO_2_.

| Day | 390 ppmv CO_2_ | 780 ppmv CO_2_ | Ratio  (780/390) | *P* |
| --- | --- | --- | --- | --- |
| Shoot DW (mg) |  |  |  |  |
| Day 10 | 0.124±0.010 | 0.200±0.011 | 1.615 | *** |
| Day 12 | 0.225±0.006 | 0.402±0.010 | 1.784 | *** |
| Day 14 | 0.503±0.017 | 0.901±0.034 | 1.794 | *** |
| Day 16 | 0.759±0.018 | 1.474±0.038 | 1.942 | *** |
| Day 18 | 1.159±0.066 | 2.363±0.144 | 2.039 | *** |
| Day 20 | 1.918±0.058 | 4.354±0.122 | 2.270 | *** |
| Day 22 | 2.738±0.107 | 7.106±0.178 | 2.595 | *** |
| Day 24 | 3.912±0.153 | 9.842±0.352 | 2.516 | *** |
| Day 26 | 4.673±0.223 | 14.130±0.629 | 3.024 | *** |
| Day 28 | 6.290±0.208 | 18.271±0.588 | 2.905 | *** |
| Day 30 | 8.053±0.341 | 22.294±0.910 | 2.768 | *** |

*** denotes statistically significant difference between plants grown at 390 and 780 ppmv CO_2_ at each day using Student’s *t*-test (*P* < 0.001). n=12-24.
