# Supplementary material for: Effects of Elevated CO2 on Levels of Primary Metabolites and Transcripts of Genes Encoding Respiratory Enzymes and Their Diurnal Patterns in Arabidopsis thaliana: Possible Relationships with Respiratory Rates
Source: Plant Cell Physiol. 2014 Jan 18;55(2):341–57. doi: 10.1093/pcp/pct185 (PMC3913440; doi:10.1093/pcp/pct185)
Supplement: Supplementary Data [file supp_pct185_pcp-2013-e-00454-File014.docx]

Table S2. Relative growth rate (RGR) of shoots of *A. thaliana* grown at 390 and 780 ppmv CO_2_.

| Day | 390 ppmv CO_2_ | 780 ppmv CO_2_ |
| --- | --- | --- |
| Day 12 | 0.350 | 0.376 |
| Day 16 | 0.209 | 0.241 |
| Day 20 | 0.215 | 0.275 |
| Day 24 | 0.134 | 0.172 |
| Day 28 | 0.136 | 0.114 |
