# Supplementary material for: Effects of Elevated CO2 on Levels of Primary Metabolites and Transcripts of Genes Encoding Respiratory Enzymes and Their Diurnal Patterns in Arabidopsis thaliana: Possible Relationships with Respiratory Rates
Source: Plant Cell Physiol. 2014 Jan 18;55(2):341–57. doi: 10.1093/pcp/pct185 (PMC3913440; doi:10.1093/pcp/pct185)
Supplement: Supplementary Data [file supp_pct185_pcp-2013-e-00454-File015.doc]

Table S3: Genes and primers used for the real-time PCR.

| Locus | Description | Primers | References |
| --- | --- | --- | --- |
| *HXK1* (At4g29130) | Hexokinase 1 | fwd 5’-TGCTGCTTTCTTTGGCGATACAGT-3' | Karve *et al.*, 2008 |
|  |  | rev 5’-CCTCGGGTTGCTATGATGTT-3’ |  |
| *G6PI* (At5g42740) | Glucose-6-phosphate | fwd 5’-GGCTATCTATGAACACAGAG-3 |  |
|  | isomerase | rev 5’-CTCGGAACTTGTCTCGAGAT-3 |  |
| *PFK7* (At5g56630) | Phosphofructokinase 7 | fwd 5’-TGGTTACACTGGCTACACCA-3 |  |
|  |  | rev 5’-TATCGACAGCTCCGTCGTCA-3 |  |
| *FBA1* (At2g21330) | Fructose 1,6- | fwd 5’-CCGGAATCATGTTCTTGTCT-3 |  |
|  | bisphosphate aldolase 1 | rev 5’-GCTGAGCCAGCGAATTGGCT-3 |  |
| *GAPC2* (At1g13440) | Glyceraldehyde-3-phosphate | fwd 5’-GGCCATCAAGGAGGAATCTG-3’ |  |
|  | dehydrogenase C2 | rev 5’-TGTGAACGATAAGGTCAACG-3’ |  |
| *ENOC* (At2g29560) | Cytosolic enolase | fwd 5’-GTGGTGACATCTCATAGATG-3’ |  |
|  |  | rev 5’-AGCTTCCAATCTTCTCCAGC-3’ |  |
| *PK* (At2g36580) | Pyruvate kinase | fwd 5’-GGACTCTAGGTCCGAAATCT-3 |  |
|  |  | rev 5’-AACTTGCAACTCAGGTCCTA-3’ |  |
| *PDH E1 alpha* (At1g59900) | Pyruvate dehydrogenase E1 | fwd 5’-CATACAGGTACCACGGTCAC-3’ |  |
|  | alpha subunit | rev 5’-GGCTCTGGCATTGGGCAATC-3’ |  |
| CSY4 (At2g44350) | mitochondrion targeted | fwd 5’-CGTGGCCAAATGTTGATGCT-3’ |  |
|  | citrate synthase 4 | rev 5’-CTTCAAGCCAGTCCATGGTA-3’ |  |
| *ACO3* (At2g05710) | Aconitase 3 | fwd 5’-CAAGCAAACATGGAGCTTGA-3’ | Yoshida & Noguchi, 2009 |
|  |  | rev 5’-AAGCTGAGGCAACAATGCTT-3’ | Yoshida & Noguchi, 2009 |
| *PEPC1* (At1g53310) | Phospho*enol*pyruvate | fwd 5’-GAGTACTTCCGCCTCGCTAC-3’ |  |
|  | carboxylase 1 | rev 5’-TGCTGATCCGAATCCAAGCC-3’ |  |
| *CI76* (At5g37510) | Complex I | fwd 5’-ACAAGGTGTGTACGATTTGC-3’ | Michalecka *et al.*, 2003 |
|  | 76 kDa subunit | rev 5’-TTTGAGGTCAAGGCTCCAAC-3’ | Michalecka *et al.*, 2003 |
| *CIII14* (At5g25450) | complex III 14 kDa subunit | fwd 5’-GAAGTCTGTTTCTAATCGCCTC-3’ |  |
|  |  | rev 5’-ACTGCCTGAAGATTATCTGG-3’ |  |
| *COX6a* (At4g37830) | Cytochrome *c* oxidase | fwd 5’-TCTTTCCCGAGCAGTGAC-3’ |  |
|  | subunit 6A | rev 5’-GATAGGCAGGAGGGTCTTCG-3’ |  |
| *ATPS* (At5g08670) | ATP synthase | fwd 5’-GATGACAAGTTGACTGTTGC-3’ |  |
|  |  | rev 5’-TGCAACCACCTCATCGATAC-3’ |  |
| *AOX1a* (At3g22370) | Alternative oxidase 1a | fwd 5’-AAGGCGGCGAAATCGCTGTT-3’ |  |
|  |  | rev 5’-TCCTCCTTCATCGGAGTTTTCTC-3’ | Huang *et al.*, 2002 |
| *NDA1* (At1g07180) | Type II NAD(P)H | fwd 5’-CTCCGTGAGAGCAAGGAAGG-3’ | Michalecka *et al.*, 2003 |
|  | dehydrogenase A1 | rev 5’-GGCGAAGTGGAGGGGATATG-3’ | Michalecka *et al.*, 2003 |
| *NDB2* (At4g05020) | Type II NAD(P)H | fwd 5’-ACTGACTCTCAAAGAGTTCC-3’ |  |
|  | dehydrogenase B2 | rev 5’-CCGATTTGAACTCTTCGATC-3’ | Clifton *et al.*, 2005 |
| *UCP1* (At3g54110) | Uncoupling protein 1 | fwd 5’-TCTGCTCTTGCTGGTGATGT-3’ | Escobar *et al.*, 2004 |
|  |  | rev 5’-TACCCAGTGCACCTGTTGTC-3’ | Escobar *et al.*, 2004 |
| *Rps15aA* (At1g07770) | 40S ribosomal protein | fwd 5’-AAGGCAAGATGGTAAGAATCAG-3’ | Watanabe *et al.*, 2008 |
|  | subunit 15aA | rev 5’-TCACATATAGAAGAAGCCGAGAACCT-3’ | Watanabe *et al.*, 2008 |
| *EF-1-a* (At5g60390) | Elongation factor 1-alpha | fwd 5’-TTGACCAGATCAACGAGCCCAAGA-3’ | Zarkovic *et.al.*, 2005 |
|  |  | rev 5’-ACTCGTGGTGCATCTCAACAGACT-3’ | Zarkovic *et.al.*, 2005 |
| *18S rRNA* (At2g01010) | 18S ribosomal RNA | fwd 5’-CTGCCAGTAGTCATATGCTT-3’ | Yoshida *et al.*, 2008 |
|  |  | rev 5’-ACTACGGTTATCCGAGTAGT-3’ | Yoshida *et al.*, 2008 |
| *ACT3* (At3g53750) | Actin 3 | fwd 5’-GGCTAACCGTGAGAAGATGA-3’ |  |
|  |  | rev 5’-CGACCTGCAAGATCAAGACG-3’ |  |
| *UBQ10* (At4g05320) | Ubiquitin 10 | fwd 5’-TGTTGCGTCTGCGTGGAGGT-3’ |  |
|  |  | rev 5’-GAAGAGTAGACTCCTTCTGG-3’ |  |

**References**

**Clifton R, Lister R, Parker KL, Sappl PG, Elhafez D, Millar AH, Day DA, Whelan J. 2005.** Stress-induced co-expression of alternative respiratory chain components in *Arabidopsis thaliana*. *Plant Molecular Biology* **58**: 193-212.

**Escobar MA, Franklin KA, Svensson S, Salter MG, Whitelam GC, Rasmusson AG. 2004.** Light regulation of the Arabidopsis respiratory chain. Multiple discrete photoreceptor responses contribute to induction of type II NAD(P)H dehydrogenase genes. *Plant Physiology* **136**: 2710-2721.

**Huang X, von Rad U, Durner J. 2002.** Nitric oxide induces transcriptional activation of the nitric oxide-tolerant alternative oxidase in Arabidopsis suspension cells. Planta 215: 914-923.

**Karve A, Rauh BL, Xia X, Kandasamy M, Meagher RB, Sheen J, Moore BD. 2008.** Expression and evolutionary features of the hexose gene family in Arabidopsis. *Planta* **228**: 411-425.

**Michalecka AM, Svensson AS, Johansson FI, Agius SC, Johanson U, Brennicke A, Binder S, Rasmusson AG. 2003.** Arabidopsis genes encoding mitochondrial type II NAD(P)H dehydrogenases have different evolutionary origin and show distinct responses to light. *Plant Physiology* **133**: 642-652.

**Watanabe CK, Hachiya T, Terashima I, Noguchi K. 2008.** The lack of alternative oxidase at low temperature leads to a disruption of the balance in carbon and nitrogen metabolism, and to an up-regulation ofantioxidant defence systems in *Arabidopsis thaliana* leaves. *Plant, Cell and Environment* **31**: 1190-1202.

**Yoshida K, Watanabe C, Kato Y, Sakamoto W, Noguchi K. 2008.** Influence of chloroplastic photo-oxidative stress on mitochondrial alternative oxidase capacity and respiratory properties: A case study with Arabidopsis *yellow variegated 2*. *Plant and Cell Physiology* **49**: 592-603.

**Yoshida K, Noguchi K. 2009.** Differential gene expression profiles of the mitochondrial respiratory components in illuminated Arabidopsis leaves. *Plant and Cell Physiology* **50**: 1449-1462.

**Zarkovic J, Anderson SL, Rhoads DM. 2005.** A reporter gene system used to study developmental expression of alternative oxidase and isolate mitochondrial retrograde regulation mutantsin Arabidopsis. *Plant Molecular Biology* **57**: 871–888.
