# Supplementary material for: Effects of Elevated CO2 on Levels of Primary Metabolites and Transcripts of Genes Encoding Respiratory Enzymes and Their Diurnal Patterns in Arabidopsis thaliana: Possible Relationships with Respiratory Rates
Source: Plant Cell Physiol. 2014 Jan 18;55(2):341–57. doi: 10.1093/pcp/pct185 (PMC3913440; doi:10.1093/pcp/pct185)
Supplement: Supplementary Data [file supp_pct185_pcp-2013-e-00454-File016.doc]

Table S4: Results of two-way analysis of variance (ANOVA) between times of day and between plants grown at 390 and 780 ppmv in Figures. *, **, and *** denote statistical significance at *P* < 0.05, 0.01, and 0.001, respectively.

|  | F_time_ | F_CO2_ | F_time×CO2_ |
| --- | --- | --- | --- |
| Fig. 1 |  |  |  |
| *HXK1* | 6.862** | 0.531 | 1.340 |
| *G6PI* | 7.103** | 0.450 | 2.090 |
| *PFK7* | 22.639*** | 1.680 | 1.630 |
| *FBA1* | 51.670*** | 6.902* | 1.431 |
| *GAPC2* | 40.973*** | 1.908 | 6.601** |
| *ENOC* | 65.501*** | 0.621 | 5.588* |
| *PK* | 2.632 (*P* = 0.099) | 0.003 | 1.713 |
| *PEPC1* | 106.929*** | 2.709 | 4.523* |
| *PDH* | 7.768** | 0.115 | 5.299* |
| *CSY4* | 60.409*** | 5.388* | 6.823** |
| *ACO3* | 17.638*** | 1.530 | 1.775 |
| Fig. 2 |  |  |  |
| *CI76* | 20.363*** | 3.470 (*P* = 0.079) | 5.768* |
| *CIII14* | 42.835*** | 11.125** | 5.536* |
| *COX6a* | 8.573** | 0.258 | 2.524 |
| *ATPS* | 19.577*** | 4.288 (*P* = 0.053) | 8.768** |
| *AOX1a* | 131.461*** | 0.548 | 0.980 |
| *NDA1* | 220.740*** | 5.240* | 2.709 |
| *NDB2* | 24.507*** | 0.001 | 3.786* |
| *UCP1* | 5.876* | 1.923 | 0.764 |
| Fig. 3 |  |  |  |
| Enolase | 8.849** | 1.930 | 0.928 |
| PEPC | 2.744 (*P* = 0.084) | 0.157 | 0.240 |
| Citrate synthase | 2.974 (*P* = 0.070) | 0.247 | 0.044 |
| Aconitase | 4.867* | 0.065 | 0.979 |
| Fumarase | 6.247** | 0.002 | 0.205 |
| Fig. 4 |  |  |  |
| Starch | 1078.679*** | 66.997*** | 11.989*** |
| Hexose-P | 113.819*** | 26.365*** | 0.468 |
| Glycolysis intermediates | 202.188*** | 9.788** | 1.030 |
| TCA-cycle intermediates | 17.843*** | 1.116 | 0.735 |
| Total amino acids | 101.341*** | 4.549* | 0.763 |
| Fig. 5 |  |  |  |
| G1P | 4.792* | 0.009 | 0.910 |
| G6P | 128.699*** | 28.195*** | 0.622 |
| F6P | 202.036*** | 28.594*** | 0.014 |
| M6P | 87.954*** | 21.493*** | 1.280 |
| GAP | 18.048*** | 4.865* | 0.314 |
| PGA | 151.685*** | 5.901* | 0.163 |
| PEP | 4.334* | 4.618* | 0.531 |
| Pyruvate | 14.391*** | 0.001 | 0.853 |
| Lactate | 0.049 | 0.001 | 0.241 |
| Citrate | 44.770*** | 0.148 | 0.754 |
| *cis*-aconitate | 6.742** | 4.273* | 0.711 |
| Succinate | 11.670*** | 4.217 (*P* = 0.051) | 0.645 |
| Fumarate | 54.235*** | 1.664 | 0.973 |
| Malate | 167.030*** | 0.407 | 0.025 |
| Fig. 6 |  |  |  |
| Glu | 2.492 | 4.812* | 0.486 |
| Gln | 373.256*** | 11.919** | 5.692** |
| Asp | 46.713*** | 25.623*** | 19.156*** |
| Asn | 46.484*** | 26.765*** | 6.999** |
| Gly | 260.643*** | 89.220*** | 26.646*** |
| Ser | 445.962*** | 2.440 | 13.113*** |
| Ala | 13.484*** | 40.721*** | 1.232 |
| Thr | 512.048*** | 21.556*** | 1.542 |
| Gly/Ser | 202.070*** | 84.402*** | 30.919*** |
| Fig. 8 |  |  |  |
| CO_2_ efflux rate | 20.953*** | 3.705 (*P* = 0.064) | 0.775 |
| O_2_ uptake rate | 159.691*** | 0.201 | 15.492*** |
| Fig. S2 |  |  |  |
| Glucose | 236.703*** | 8.435** | 8.957** |
| Sucrose | 131.453*** | 0.122 | 0.558 |
| Fig. S3 |  |  |  |
| Val | 40.145*** | 3.346 (*P* = 0.080) | 4.507* |
| Leu | 22.982*** | 2.449 | 3.103 |
| Lys | 95.114*** | 0.366 | 14.900*** |
| Met | 0.957 | 6.680* | 1.207 |
| Ile | 38.972*** | 0.414 | 2.584 |
| Trp | 11.589*** | 0.111 | 2.110 |
| Tyr | 9.953** | 1.462 | 0.609 |
| Phe | 73.044*** | 0.060 | 1.186 |
| Pro | 22.297*** | 0.684 | 4.198* |
| Cir | 321.977*** | 1.264 | 2.019 |
| Arg | 17.852*** | 5.348* | 14.672*** |
| His | 48.194*** | 1.342 | 2.619 |
